# Overall Prevalence and Prevalence Compared among Psoriasis Treatments of Onychomycosis in Patients with Nail Psoriasis and Fungal Involvement

**DOI:** 10.1155/2021/9113418

**Published:** 2021-12-13

**Authors:** Leena Chularojanamontri, Penvadee Pattanaprichakul, Charussri Leeyaphan, Panittra Suphatsathienkul, Supisara Wongdama, Sumanas Bunyaratavej

**Affiliations:** Department of Dermatology, Faculty of Medicine Siriraj Hospital, Mahidol University, Bangkok, Thailand

## Abstract

**Background:**

Whether nail psoriasis can increase the risk of onychomycosis is still being debated, and data relating to the prevalence of onychomycosis among psoriasis patients receiving different treatments is limited.

**Objectives:**

To investigate the overall prevalence and prevalence compared among psoriasis treatments of onychomycosis in patients with nail psoriasis and fungal involvement.

**Methods:**

A prospective study of three groups of nail psoriasis being treated with only topical medication, methotrexate, or biologics (25 patients per group, 150 nails) was conducted at Siriraj Hospital (Bangkok, Thailand) during November 2018 to September 2020. Demographic data, psoriasis severity, and nail psoriasis severity were recorded. The nail most severely affected with psoriasis on each hand was selected for mycological testing. Potassium hydroxide, periodic acid-Schiff stain, and fungal culture were performed.

**Results:**

The prevalence of onychomycosis in nail psoriasis was 35.3%. Among the treatment groups, the prevalence of onychomycosis was significantly higher in the methotrexate group than in the topical treatment and biologic treatment groups (*p* = 0.014). *Candida* spp. was the main causative organism, followed by *Trichophyton rubrum*. Thumb was most commonly affected (59.3%). The most common abnormality of the nail matrix and the nail bed was pitted nail (71.3%) and onycholysis (91.3%), respectively. Multivariate analysis revealed diabetes, wet-work exposure, and methotrexate treatment to be predictors of onychomycosis.

**Conclusions:**

Several factors, including psoriasis treatment, were shown to increase the risk of onychomycosis in nail psoriasis. Further research is needed to determine whether biologic agents, especially interleukin-17 inhibitors, can increase risk of onychomycosis and *Candida* infection/colonization of the nails.

## 1. Introduction

Psoriasis is a multifactorial chronic disorder that has an etiopathogenesis that derives from the alteration of signaling pathways, which leads to a defect in the functional and structural properties of the skin [1]. It can cause nail pathology on both the hands and feet. The reported prevalence of nail involvement in patients with psoriasis varied considerably from 15% to 86% [2, 3]. The presence of nail involvement was reported to be a predictor of psoriatic arthritis, and it was also found that nail involvement may occur a few years before the development of joint disease [4]. Psoriasis can affect the nail matrix and nail bed resulting in several clinical presentations, including pitting, crumbling, onycholysis, and subungual hyperkeratosis [5].

Onychomycosis is a fungal nail infection that is caused by dermatophytes, yeasts, and filamentous fungi. It is a common nail disease that accounts for 50% of nail disorders. Its prevalence in the general population and in patients with psoriasis can reach up to 30% and 56%, respectively [5, 6]. Clinical features of nail psoriasis and onychomycosis may overlap, and pathologies of both diseases may occur in the same patient. Several factors, including nail pathologies, patient behavior, immune status, and treatments for psoriasis, may contribute to the development of onychomycosis in nail psoriasis.

Topical treatments, such as steroids, vitamin D3 analogs, tazarotene, trifarotene, topical calcineurin inhibitors, and 5-fluorouracil, can be used to treat nail psoriasis [7–9]. For patients with severe skin involvement and nail psoriasis, conventional systemic treatments (ciclosporin, methotrexate, and acitretin), small molecule drugs, and biologics are recommended. Due to their immunosuppressive properties, ciclosporin, methotrexate, and biologics may aggravate onychomycosis in nail psoriasis [3]. On the other hand, there is evidence that vitamin A and its active metabolite, all-transretinoic acid, exert host-protective effects in infections and direct fungistatic effect against *Candida albicans* [9–11]. Recently, small molecule drugs, such as apremilast and tofacitinib, have been shown to be effective for treating nail psoriasis [3, 12].

Although several studies have investigated the prevalence of onychomycosis in patients with nail psoriasis, few studies have addressed its prevalence among patients receiving different treatments for psoriasis. Among those studies, a cross-sectional study reported a prevalence of onychomycosis of 34.8% (8/23) among patients not being treated with immunosuppressive agents, and yeasts, and filamentous fungi were the predominant pathogens [13]. Other studies reported that factors affecting the immune status, including diabetes, administration of topical corticosteroids, and systemic treatments for psoriasis, were risk factors for onychomycosis in patients with psoriasis [6, 14]. A randomized prospective open-label study reported that the risk of onychomycosis in psoriasis patients receiving treatment with an antitumor necrosis factor (anti-TNF) was 20.3% compared to 13.9% in patients that did not receive any biological agents [15]. Moreover and importantly, published data relating to the prevalence of onychomycosis among patients receiving anti-interleukin- (IL-) 17, in which mucocutaneous candidiasis is a side effect of concern, is comparatively scarce [16].

Thus, the aim of this study was to investigate the overall prevalence and prevalence of onychomycosis among different treatments for psoriasis, including topical medication, methotrexate, or biologic therapy in patients with nail psoriasis. Our secondary objective was to identify significant risk factors for developing onychomycosis in this patient population.

## 2. Materials and Methods

This prospective study was conducted at the Department of Dermatology, Faculty of Medicine Siriraj Hospital, Mahidol University, Bangkok, Thailand during November 2018 to September 2020. The inclusion criteria were psoriasis patients with fingernail pathologies aged 18 years or older who attended the outpatient dermatology clinic at our center and who were being treated with only topical treatment, methotrexate, or biologic agents for at least four weeks. The exclusion criteria were (i) patients with a history of receiving any topical or systemic antifungal agents during the 12-week period prior to the start of this study, (ii) patients taking other immunosuppressant drugs, (iii) pregnant or lactating patients, and/or (iv) patients with other nail diseases. All patients who voluntarily agreed to participate in this study provided written informed consent. The protocol for this study was approved by the Siriraj Institutional Review Board (SIRB) (COA no. Si.844/2018).

Demographic and clinical data, including gender, age, body mass index (BMI), duration of psoriasis, underlying diseases, psoriasis type, having or not having psoriatic arthritis, occupation, dominant hand side, and handwashing frequency, were recorded. Current psoriasis severity was assessed using the Psoriasis Area and Severity Index (PASI). The most severely affected fingernail of each hand was assessed using the Nail Psoriasis Severity Index (NAPSI) and the Nijmegen-Nail Psoriasis Activity Index tool (N-NAIL) [16, 17]. For NAPSI, the nail was divided into four quadrants, after which each quadrant was evaluated for the presence or absence of any of eight parameters indicating nail bed and/or nail matrix pathologies. The NAPSI score for one fingernail ranged from 0 to 8 [16]. For N-NAIL, the five evaluated parameters include onycholysis/oil drop, pitting, crumbling, Beau lines, and subungual hyperkeratosis. A score of 0 indicates no nail involvement, whereas a score of 3 represents maximum severity for each parameter for a total possible score of 15 for each fingernail [17]. The skin around each fingernail was evaluated for paronychia, and each nail was evaluated, as follows: grade 1 = redness and swelling of the nail folds; grade 2 = pronounced redness and swelling of the nail folds; grade 3 = redness and swelling of the nail folds with no cuticle; grade 4 = redness and swelling of the nail folds, no cuticle, and tenderness and pain; and grade 5 = grade 4 plus acute paronychia on top of chronic paronychia [18].

Specimens obtained from the distal part of the most severely affected psoriatic nail from each hand were tested for onychomycosis using potassium hydroxide (KOH), nail clipping for periodic acid-Schiff stain (PAS) technique, and fungal culture on Sabouraud dextrose agar and chloramphenicol media with and without cycloheximide (HiMedia Laboratories, Mumbai, India). Specimen collection and interpretation of KOH examination, PAS stain, and fungal cultures were performed by experienced technicians and one dermatopathologist. Cultures were incubated at 30°C and examined weekly up to 4 weeks. Chromogenic Candida Agar (Oxoid, Basingstoke, UK) was used to identify *C. tropicalis*, *C. krusei*, *C. albicans*, and *C. dubliniensis* on the basis of the morphology and color of the colonies [19]. Blue and brown/pink colonies indicate *C. tropicalis* and *C. krusei*, respectively. Green colonies indicate *C. albicans* or *C dubliniensis*. The ability to grow at 42°C differentiates *C. albicans* (growth) from *C. dubliniensis* (no growth) [20]. Other *Candida* species that developed natural, mauve, or rose colors would be referred to *Candida* spp. in this study.

Specimens were collected from the nail bed as proximally to the cuticle as possible using a scalpel blade. Onychomycosis was diagnosed if (i) culture was positive for pathologic fungi whether direct microscopy (KOH and PAS) was positive or negative or if (ii) culture was negative, but PAS stain revealed pathological forms of fungal infection whether direct KOH examination was positive or negative. Fungal colonization of the nail was diagnosed if culture was positive for *Candida* species, but direct microscopy was negative. If the results were negative for all three techniques (KOH, PAS, culture), negative pathogen would be diagnosed [21]. Patients with a diagnosis of onychomycosis were defined as patients who had onychomycosis of the right and/or left hand. KOH examination and culture were also performed at the proximal nail fold. *Candida* infection of the nail fold was diagnosed if there was at least grade one of paronychial involvement and positive result for both direct microscopy (KOH) and culture. *Candida* infection in the oral cavity was also recorded. Oral candidiasis was diagnosed if both KOH and culture showed a positive result for *Candida* infection.

### 2.1. Sample Size Calculation and Statistical Analysis

The sample size for each group was calculated using *λ*^2^. Previous studies reported the prevalence of onychomycosis in psoriasis patients treated with topical medication, methotrexate, and biologic agents to be 20%, 50%, and 13%, respectively [14, 15, 21]. Using a 2-sided type I error of 0.05 and 80% power, a sample of 25 patients per group was required (75 patients in total).

Descriptive statistics were used to summarize patient demographic and clinical characteristics. Data are described as mean plus/minus standard deviation (SD) for continuous data with normal distribution and as median and interquartile range (IQR) for nonnormally distributed continuous data. Categorical data are described as number and percentage. The results of univariate analysis and multivariate analysis are shown as odds ratio and adjusted odds ratio with their 95% confidence intervals, respectively. Statistical Package for the Social Sciences 18.0 (SPSS, Inc., Chicago, IL, USA) was used for data analysis, and a *p* value less than 0.05 was considered to be statistically significant.

## 3. Results

Among the 75 enrolled patients, the gender distribution was almost equal, and the mean age was 46.8 ± 15.3 years. The most common underlying disease was dyslipidemia (24.0%), followed by hypertension (22.7%), diabetes mellitus (18.7%), metabolic syndrome (13.3%), and obesity (12.0%).  Table 1 shows the demographic and clinical characteristics of the included patients. Most patients had psoriasis without psoriatic arthritis (82.7%). Forty patients (53.3%) had an occupation that did not expose the patient to an increased risk of hand exposure to water. The median handwashing frequency of patients was 6 times/day, with a minimum of 2 times/day and a maximum of 20 times/day. Patterns of nail involvement and nail pathology were similar between the right hand and the left hand. Thirteen patients (17.3%) had oral candidiasis. We also found significant correlation between oral candidiasis and *Candida* infection of the nails (*p* = 0.015, data not shown).

**Figure 1:**
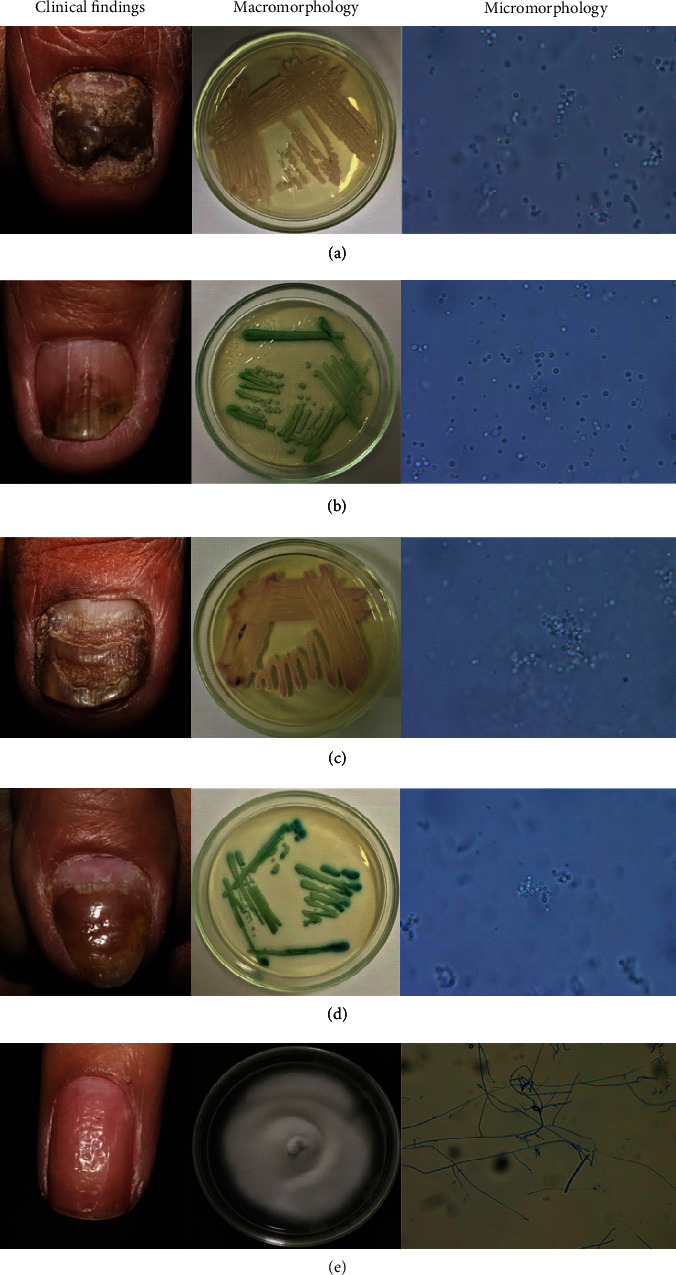
Representative pictures of clinical findings of psoriatic nail with fungal infection. Culture and microscopic identification from specimens collected from the distal part of psoriatic nail were also demonstrated. The fungi were inoculated onto the Chromogenic Candida Agar (a–d) and the Sabouraud dextrose agar with chloramphenicol (e) and inoculated at 30°C for 4 weeks. (a) *Candida* spp., (b) *C. albicans*, (c) *C. krusei*, (d) *C. dubliniensis*, and (e) *Trichophyton rubrum*.

Of 150 fingernails (1 from each hand of each of 75 patients), the thumb was the most severely affected fingernail on both hands. Pitted nail and onycholysis were the most common abnormalities of the nail matrix and nail bed, respectively. Seventy-three fingernails had paronychial involvement with 71.2%, 26.0%, and 2.7% of grades 1, 2, and 3 paronychial involvement, respectively. Fifty-three (35.3%) fingernails and 34 (22.7%) fingernails had onychomycosis and *Candida* colonization, respectively (Table 2). The most common organism causing onychomycosis was *Candida* spp., followed by *Trichophyton rubrum*. *Candida* infection of the nail fold was found in 13 (17.8%) of the 73 fingernails with paronychial involvement. The photos of clinical findings of nail psoriasis with fungal infection and macro and micromorphology of the fungal isolates are shown as Figure 1.

Univariate analysis showed concomitant diabetes, occupation that might increase the risk of hand exposure to water, and methotrexate to be significant risk factors associated with onychomycosis (Table 3), and all three of those factors remained statistically significant in multivariate analysis. When we excluded patients with diabetes and reanalyzed our data, wet-work exposure and methotrexate were significant risk factors for the development of onychomycosis in univariate analysis (*p* = 0.002, *p* = 0.028), and wet-work exposure remained as the only risk factor independently associated with onychomycosis in multivariate analysis (*p* = 0.007).  Table 4 shows that the prevalence of onychomycosis was highest in the methotrexate group (68% in methotrexate treatment, 40% in topical treatment, and 28% in biologic treatment). However, the number of patients with diabetes and wet-work exposure was higher in the methotrexate group than in the other two groups. The risk of *Candida* colonization of the nail did not increase significantly in the biologic treatment group even though the main biologics used were interleukin-17 inhibitors.

## 4. Discussion

A systematic review in 2014 showed an increased prevalence of onychomycosis in psoriatic patients (18%) compared to the prevalence in the general population (9.1%); however, the high heterogeneity among the 10 included studies limits the reliability of their findings [22]. We reviewed the literature in the PubMed database using the keywords “*prevalence*,” “*onychomycosis*,” “*psoriasis*,” and “*treatment*.” Thirty studies were included and summarized, as shown in Table 5 [2, 6, 13–15, 21, 23–46]. It was shown that the prevalence of onychomycosis in psoriatic nails in Asian countries ranged from 20.3% (Kuwait) to 47.9% (India) compared to the prevalence of 18.0% (Belgium, Poland) to 62.0% (Bulgaria) in European countries [2, 6, 13–15, 23, 25, 27–33, 35, 37, 40, 41, 44–46]. The prevalence of onychomycosis in controls with clinical abnormality in Asian countries ranged from 4% (Pakistan) to 40.6% (Turkey) compared to the prevalence of 22.4% (Poland) to 51.3% (Italy) in European countries [2, 14, 15, 32, 33, 35, 37, 44–46]. The first study of the prevalence of onychomycosis in psoriasis patients at our center was published in 2018, and that study found a prevalence of 32.3% [23]. The prevalence in this study was 35.3% (53/150), which is close to, but higher than that from the previous study. It seemed that the prevalence of onychomycosis in psoriatic patients in the present study was in a range of the reported prevalence of onychomycosis in controls with clinical nail abnormality in Asian countries [15, 35, 45].

Yeasts (*Candida* spp.) were more commonly identified in the present study, which is similar to several previous studies [2, 13,14, 23, 24]. Chadeganipour et al. used molecular technique to identify *Candida* species in clinical samples of patients with psoriasis [47]. Molecular technique can provide 95% sensitivity and 100% specificity [48]. In that study, *C. parapsilosis* was the most prevalent species among *Candida* species of fingernail infection and none of *Candida* species were albicans. A lower number of clinical samples and different technique to identify *Candida* species in Chadeganipour's study may explain the results different from our study [47].

Our study showed diabetes, methotrexate treatment, and wet-work exposure to be significant risk factors associated with onychomycosis. Methotrexate was at greater risk of developing onychomycosis compared to biologic treatment. The pathogenesis of nail psoriasis may increase or decrease the risk of onychomycosis. Generally, rapid nail growth, increase in antimicrobial peptides, and compact orthokeratotic nail plate will decrease the risk of onychomycosis in nail psoriasis [5]. However, other factors that increase the risk of onychomycosis also play an important role. Methotrexate can increase the risk of onychomycosis by immunosuppressive effect and slow the rate of nail growth [6].

Studies reporting the prevalence of onychomycosis in psoriasis patients who were on biologic treatment mainly included patients receiving anti-TNF treatment [14, 15, 21, 24]. Three out of four studies, including a randomized prospective study, showed a higher prevalence of onychomycosis in psoriatic patients treated with anti-TNF than in patients that received other types of treatments [14, 15, 21, 24]. There are some possible explanations why biologics did not increase the risk of onychomycosis in our study. First, the faster nail growth rate in psoriasis [49–51] acts as a protective factor against onychomycosis. Second, biologic treatment is more effective for treating nail psoriasis than methotrexate [52] because it reduces nail pathology and the opportunity of the fungi to invade the nail keratin. Third and last, the biologics most often used in our study were interleukin-17 inhibitors, which may have less immunosuppressive effect than anti-TNF.

### 4.1. Limitations

This study has some mentionable limitations. Even though we enrolled a sufficient number of patients to satisfy the minimum described by our sample size calculation, 75 patients represent a relatively small number of patients. Besides, only 20 patients were treated with interleukin-17 inhibitors, which could limit the generalizability of our results. Secondly, toenails were not included in this study since several other factors influence the risk of developing onychomycosis of the toenails compared to the fingernails. Thirdly, in cases of diagnostic discordance among the 3 diagnostic methods, a retest was not performed. Our rationale for this is that all three diagnostic methods were performed by experienced technicians and one expert dermatopathologist. Finally, molecular biology testing, which provides high sensitivity, high specificity, and accurate identification of fungal species, could not be performed in this study due to its relatively high cost. Alternatively, our laboratory used a culture-based method to identify fungal species. Only *C. albicans*, *C. dubliniensis*, *C. tropicalis*, and *C. krusei* could be identified. This is why we reported the other groups of *Candida* as *Candida* spp.

## 5. Conclusions

The prevalence of onychomycosis in nail psoriasis in this study was 35.3%. Among the three different psoriasis treatment groups, the prevalence of onychomycosis was significantly higher in the methotrexate treatment group than in the topical treatment and biologic treatment groups. Several factors can affect the risk of onychomycosis, including occupation, psoriasis treatment, host status, and nail growth rate. Molecular identification would be the best method to elucidate the etiology and establish an epidemiological inference with previous findings in the literature.

## Figures and Tables

**Table 1:** Demographic and clinical characteristics of nail psoriasis patients with onychomycosis.

Characteristics (*N* = 75)	Values
Gender, *n* (%)	
Female	38 (50.7%)
Male	37 (49.3%)
Age (years), mean ± SD	46.8 ± 15.3
Body mass index (kg/m^2^), mean ± SD	25.0 ± 5.0
Duration of psoriasis (years), median (IQR)	11.0 (7.0, 20.0)
Psoriasis types, *n* (%)	
Plaque	64 (85.3%)
Guttate	7 (9.3%)
Erythrodermic	3 (4.0%)
Pustular	1 (1.3%)
Psoriatic arthritis, *n* (%)	13 (17.3%)
Occupation, *n* (%)	
No increased risk of hand exposure to water^†^	40 (53.3%)
Increased risk of hand exposure to water^‡^	35 (46.7%)
Patient right-handed, *n* (%)	68 (90.7%)
Patient left-handed, *n* (%)	7 (9.3%)
Handwashing frequency (times/day), median (IQR)	6.0 (4.0, 10.0)
Current psoriasis severity	
Psoriasis Area and Severity Index score, median (IQR)	5.0 (3.2, 10.2)
Characteristics of fingernail pathologies (*N* = 150)	Values
Nail severity	
Nail Psoriasis Severity Index score (0-8), median (IQR)	4.0 (2.0, 4.0)
Nijmegen-Nail Psoriasis Activity Index tool (0-15), median (IQR)	4.0 (3.0, 5.0)
Most severely affected fingernail, *n* (%)	
Thumb	89 (59.3%)
Index finger	22 (14.7%)
Middle finger	16 (10.7%)
Ring finger	13 (8.7%)
Little finger	10 (6.7%)
Nail matrix pathology, *n* (%)	
Pitting	107 (71.3%)
Leukonychia	69 (46.0%)
Crumbling	55 (36.7%)
Red spots lunula	2 (1.3%)
Nail bed pathology, *n* (%)	
Onycholysis	137 (91.3%)
Subungual hyperkeratosis	53 (35.3%)
Oil drop	30 (20.0%)
Splinter hemorrhage	29 (19.3%)
Beau lines, *n* (%)	19 (12.7%)
Paronychial involvement, *n* (%)	73 (48.7%)

^†^Driver, lawyer, merchant, collegian, teacher, or retired. ^‡^Housekeeper, farmer, fisherman, mechanic, builder, or barber. Abbreviations: SD: standard deviation; IQR: interquartile range.

**Table 2:** Results of potassium hydroxide (KOH), periodic acid-Schiff (PAS) stain, and culture techniques.

**Specimens collected from distal part of fingernails (** **N** = 150**)**					
**Direct microscopy**		**Culture**	**Interpretation**	**n** **(%)**	**Pathogen**
**KOH testing** ^**¶**^	**PAS stain** ^**§**^				
—	—	No growth	No pathogen	62 (41.3%)	
+	—	No growth	Discordant results	1 (0.7%)	
—	—	Candida	Colonization	34 (22.7%)	(i) *Candida* spp. (*n* = 24) (ii) *C. albicans* (*n* = 5) (iii) *C. krusei* (*n* = 5)
—	+	No growth		7 (4.7%)	
+	+				
+	—		Onychomycosis	46 (30.7%)	*Candida* spp. (*n* = 25) *C. albicans* (*n* = 15) *C. krusei* (*n* = 3) *C. dubliniensis* (*n* = 2) *T. rubrum* (*n* = 1)
—	+	Pathologic fungi			
+	+				

**Specimens collected from proximal part of fingernails that had at least grade 1 of paronychial involvement (** **N** = 73**)**					
**KOH testing** ^**¶**^		**Culture**	**Interpretation**	**n** **(%)**	**Pathogen**
—		—	No pathogen	52 (71.2%)	
Pseudohyphae with budding yeast		Candida	Candida infection at the nail fold	13 (17.8%)	*Candida* spp. (*n* = 9) *C. albicans* (*n* = 2) *C. krusei* (*n* = 1) *C. dubliniensis* (*n* = 1)

^¶^KOH: + indicates pseudohyphae with budding yeast or septate hyphae. ^§^PAS: + indicates the presence of septate hyphae invading the nail plate or thicker tortuous wall hyphae or pseudohyphae with budding yeast.

**Table 3:** Analysis for risk factors independently associated with onychomycosis in patients with nail psoriasis.

	Univariate analysis		Multivariate analysis	
	Crude odds ratio (95% confidence interval)	*p* value	Adjusted odds ratio (95% confidence interval)	*p* value
Male gender	0.68 (0.27-1.70)	0.411		
Concomitant diabetes mellitus	6.06 (1.53-24.02)	***0.010***	7.04 (1.44-34.50)	***0.016***
Duration of psoriasis ≥10 years	0.99 (0.37-2.70)	0.989		
Handwashing frequency ≥ 6 times/day	1.83 (0.73-4.58)	0.200		
Occupation that increased the risk of hand exposure to water	5.05 (1.89-13.52)	***0.001***	4.12 (1.36-12.51)	***0.012***
Nail Psoriasis Severity Index score ≥ 4	1.30 (0.52-3.24)	0.570		
Nijmegen − Nail Psoriasis Activity Index score ≥ 4	1.70 (0.65-4.46)	0.281		
Topical treatment: methotrexate	3.19 (1.00-10.17)	***0.050***	2.12 (0.55-8.16)	0.275
Biologic^#^	0.58 (0.18-1.91)	0.372	0.46 (0.12-1.86)	0.277
Biologic treatment^#^: topical	1.71 (0.53-5.60)	0.372	2.16 (0.54-8.65)	0.277
Methotrexate	5.46 (1.63-18.36)	***0.006***	4.57 (1.11-18.93)	***0.036***
Pitted nails	1.51 (0.54-4.23)	0.434		
Leukonychia	1.14 (0.46-2.83)	0.785		
Crumbling	1.69 (0.65-4.41)	0.282		
Onycholysis	0.46 (0.10-2.07)	0.311		
Subungual hyperkeratosis	1.09 (0.43-2.77)	0.850		
Paronychial involvement	0.90 (0.36-2.25)	0.817		
*Candida* infection in the oral cavity	3.33 (0.92-12.01)	0.066	2.82 (0.59-13.39)	0.192

Variables with a *p* value < 0.20 in univariate analysis were included in multivariate analysis. A *p* value < 0.05 in multivariate analysis was considered statistically significant. ^#^Interleukin- (IL-) 17 inhibitors, antitumor necrosis factor, and anti-IL 12/23 were used in 20, 4, and 1 patient, respectively.

**Table 4:** Characteristics of nail psoriasis patients compared among psoriasis treatment regimens.

	**Number of patients (** **N** = 75**)**			
**Characteristics**	**Topical treatment (** **n** = 25**)**	**Methotrexate (** **n** = 25**)**	**Biologic agents** ^**#**^ **(** **n** = 25**)**	**p** **value**
Gender, *n* (%)				
Female	9 (36.0%)	13 (52.0%)	16 (64.0%)	
Male	16 (64.0%)	12 (48.0%)	9 (36.0%)	
Age (years), mean ± SD	48.1 ± 17.2	47.8 ± 11.3	44.4 ± 16.8	0.403
Body mass index (kg/m^2^), mean ± SD	23.6 ± 4.0	25.6 ± 5.4	25.9 ± 5.2	***0.042***
Concomitant diabetes mellitus, *n* (%)	2 (8.0%)	7 (28.0%)	5 (20.0%)	0.226
Occupation, *n* (%)				
Increased risk of hand exposure to water	9 (36.0%)	17 (68.0%)	9 (36.0%)	***0.032***
Nail severity, median (IQR)				
Nail Psoriasis Severity Index score	3.0 (2.0, 4.0)	3.0 (2.0, 4.0)	4.0 (2.0, 4.0)	0.979
Nijmegen-Nail Psoriasis Activity Index score	4.0 (3.0, 5.0)	4.0 (3.0, 5.0)	4.0 (3.0, 5.0)	0.333
Patients with diagnosis of onychomycosis				
No	15 (60.0%)	8 (32.0%)	18 (72.0%)	***0.014***
Yes (right or left hand)	10 (40.0%)	17 (68.0%)	7 (28.0%)	

	**Number of fingernails (** **N** = 150**)**			
**Characteristics**	**Topical treatment (** **n** = 50**)**	**Methotrexate (** **n** = 50**)**	**Biologic agents** ^**#**^ **(** **n** = 50**)**	**p** **value**
*Candida* infection of the nails	16 (32.0%)	25 (50.0%)	9 (18.0%)	***0.030***
*Candida* colonization of the nails	11 (22.0%)	8 (16.0%)	15 (30.0%)	0.245
*Candida* infection of the nail folds	7 (14.0%)	8 (16.0%)	6 (12.0%)	0.847
*Candida* infection of the nails and nail folds	7 (14.0%)	7 (14.0%)	6 (12.0%)	1.000

^#^Interleukin- (IL-) 17 inhibitors, antitumor necrosis factor, and anti-IL 12/23 were used in 20, 4, and 1 patient, respectively. A *p* value < 0.05 indicates statistical significance. Abbreviations: SD: standard deviation; IQR: interquartile range.

**Table 5:** Published literature in the PubMed database relating to the prevalence of onychomycosis in nail psoriasis.

Authors (year, country)	Patients : Controls	Fungal tests	Prevalence of onychomycosis (%)					Prevalence of onychomycosis among different treatments of psoriasis (%)			Organisms from psoriatic nails (%)
			Overall	Psoriasis patients		Controls		Topical	Systemic	Biologics	
				No clinical	Clinically abnormal	No clinical	Clinically abnormal				
Toenails											
1. Gupta et al. (1997, Canada and USA)	561 : 922	KOH+C/S	15.3	12.7 (38/298)	27 (71/263)	6.9 (54/776)	43.8 (64/146)	—	—	—	(i) DMPs 84.9 (ii) Moulds 9.4 (iii) Yeasts 5.7
2. Hamnerius et al. (2004, Sweden)	239 : 245	KOH+C/S	3.5	4.6 (11/239)	—	2.4 (6/245)	—	—	—	—	—
3. Zawirska et al. (2006, Poland)	70 : 60	KOH+C/S	9	11.4 (8/70)	—	3.3 (2/60)	—	—	—	—	—
4. Piérard-Franchimont et al. (2006, Belgium)	233 : 0	C/S+PAS	18.0	—	18.0 (42/233)	—	—	—	—	—	(i) DMPs 54.3 (ii) Yeasts 37.1 (iii) Moulds 8.6
5. Leibovici et al. (2008, Israel)	113 : 102	KOH+C/S	38.6	—	47.6 (54/113)	28.4 (29/102)	—	—	—	—	(i) DMPs 77.8 (ii) Yeasts 11.1 (iii) Moulds 11.1
6. Altunay et al. (2009, Turkey)	60 : 60	KOH+C/S	8.3	8.3 (5/60)		8.3 (5/60)		—	—	—	(i) DMPs 100
7. Vender et al. (2016, Canada)	12 : 0	KOH+C/S	25	—	25 (3/12)	—	—	—	—	—	(i) Moulds 33.3 (ii) No growth 66.7
Toenails and fingernails											
8. Staberg et al. (1983, Denmark)	78 : 41	KOH+C/S	25.2	26.9 (10/39)	30.8 (12/39)	19.5 (8/41)	—	—	—	—	(i) DMPs 47.6 (ii) Yeasts 47.6 (iii) Moulds 4.8
9. Szepes (1986, Hungary)	137 : 341	C/S	64.4	63.1 (83/137)		66.0 (225/341)		—	—	—	(i) Moulds 50.0 (ii) Yeasts 37.1 (iii) DMPs 12.9
10. Stander et al. (2001, Germany)	250 : 102	KOH+C/S	27.3	30.4 (76/250)		19.6 (20/102)		—	—	—	(i) DMPs 28.9 (ii) Yeasts 62.2 (iii) Moulds 7.9
11. Salomon et al. (2003, Poland)	106 : 0	KOH+C/S	13.8	Not tested (*n* = 23)	18 (15/83)	—	—	—	—	—	(i) Moulds 37.5 (ii) DMPs 31.3 (iii) Yeasts 31.3
12. Larsen et al. (2003, Denmark)	79 : 142	KOH+C/S	15.8	Not tested (*n* = 14)	26.2 (17/65)	Not tested (*n* = 89)	34.0 (18/53)	—	—	—	(i) Yeasts 45.5 (ii) DMPs 36.4 (iii) Moulds 18.2
13. Kacar et al. (2006, Turkey)	168 : 164	KOH+C/S	10.5	Not tested (*n* = 91)	28.6 (22/77)	Not tested (*n* = 132)	40.6 (13/32)	—	—	—	(i) DMPs 36.4 (ii) Yeasts 13.6 (iii) Moulds 9.1 (iv) No growth 40.9
14. Pawlaczyck et al. (2007, Poland)	481 : 3,986	KOH+C/S	36.3	6.0 (20/327)	18.8 (29/154)	—	39.6 (1579/3986)	—	—	—	(i) DMPs 65.5 (ii) Yeasts 27.6 (iii) Moulds 6.9
15. Sánchez-Regaña et al. (2007, Spain)	20 : 0	KOH+C/S	30	—	30 (6/20)	—	—	—	—	—	(i) Yeasts 66.7 (ii) Moulds 33.3
16. Shemer et al. (2009, Israel)	312 : 0	KOH+C/S	34.3	—	34.3 (23/67)	—	—	—	—	—	(i) DMPs 74.0 (ii) Moulds 39.1 (iii) Yeasts 30.4
17. Natarajan et al. (2010, India)	72 : 0	KOH+C/S+PAS	31.9	Not tested (*n* = 24)	47.9 (23/48)	—	—	—	—	—	(i) Moulds 50 (ii) Yeasts 50
18. Kavaliauskiene et al. (2010, Lithuania)	30 : 529	KOH+C/S	23.6	—	23.3 (7/30)	—	23.6 (125/529)	—	—	—	(i) DMPs 71.4 (ii) Yeasts 28.6
19. Zisova et al. (2011, Bulgaria)	228 : 0	KOH+C/S	62	—	62 (141/228)	—	—	—	—	—	(i) DMPs 67 (ii) Yeasts 24 (iii) Moulds 6
20. Rizzo et al. (2013, Italy)	31 : 274	C/S+PAS	37.7	—	41.9 (13/31)	—	37.2 (102/274)	—	—	—	(i) Yeasts 69.2 (ii) DMPs 30.8
21. Al-Mutairi N et al. (2013, Kuwait)	315 : 180	KOH+C/S	18.0	—	20.3 (64/315)	—	13.9 (25/180)	13.9	—	Anti-TNF 20.3 (i) IFX 33.0 (ii) ETA 15.5 (iii) ADA 13.3	(i) DMPs 65.6 (ii) Yeasts 28.1 (iii) Moulds 6.3
22. Mendez-Tovar et al. (2015, Mexico)	150 : 0	KOH+C/S	28	Not tested (*n* = 67)	50.6 (42/83)	—	—	21.4	MTX, CsA 31	**Anti-TNF** 11.9	(i) Yeasts 50 (ii) DMPs 32 (iii) Moulds 18
23. Tsentemeidou et al. (2017, Greece)	23 : 0	KOH+C/S	34.8	—	34.8 (8/23)	—	—	34.8	—	—	(i) Yeasts 37.5 (ii) Moulds 37.5 (iii) DMPs 12.5
24. Zander et al. (2017, Germany)	2781 : 136,137	Not specified	6.4	7.8 (219/2781)		6.4 (8678/136,137)		—	—	—	—
25. Romaszkiewicz et al. (2018, Poland)	102 : 2335	KOH+C/S	22.4	—	23.5 (24/102)	5 (5/100)	22.4 (520/2325)	25	MTX 20.8 CsA 12.5 ACT 4.1	**Anti-TNF** 37.5	(i) Yeasts 50.0 (ii) DMPs 29.2 (iii) Moulds 20.8
26. Chaowattanapanit et al. (2018, Thailand)	62 : 0	C/S	32.3	—	32.3 (20/62)	—	—	—	—	—	(i) Yeasts 41.9 (ii) Moulds 19.4 (iii) No growth 35.5
27. Tabassum et al. (2019, Pakistan)	159 : 318	KOH+C/S	14	—	34 (54/159)	—	4 (13/318)	34	—	—	(i) Yeasts 37.0 (ii) Moulds 35.2 (iii) DMPs 7.4
28. Jendoubi et al. (2019, Tunisia)	163 : 0	KOH+C/S	33.7	Not tested (*n* = 47)	47.4 (55/116)	—	—	—	—	—	Fingernail: Yeasts 100 Toenail: DMPs 100
29. Gallo et al. (2019, Italy)	711 : 8670	KOH+C/S	51.1	—	49.1 (349/711)	—	51.3 (4397/8570)	—	—	—	(i) Yeasts 43.6 (ii) DMPs 43.3 (iii) Moulds 13.2
30. Alves et al. (2020, Brazil)	38 : 0	KOH+C/S+PAS	57.9	—	57.9 (22/38)	—	—	33.0	MTX 92.8 ACT 33.3	**Anti-TNF** 50 **Anti-IL17** 0 **Anti-IL23** 0	(i) DMPs 56.3 (ii) Yeasts 43.8
Fingernails											
31. The present study	75 : 0 (150 nails)	KOH+C/S+PAS	36	—	36 (54/150)	—	—	44.0	MTX 68.0	**Anti-TNF** 50 **Anti-IL17** 25 **Anti-IL12/23** 0	(i) Yeasts 83.3 (ii) DMPs 1.9 (iii) No growth 14.8

Abbreviations: KOH: potassium hydroxide examination; C/S: culture; PAS: Periodic-Schiff stain; MTX: methotrexate; ACT: acitretin; CsA: ciclosporin; anti-TNF: antitumor necrosis factor; IFX: infliximab; ETA: etanercept; ADA: adalimumab; IL: interleukin; DMPs: dermatophytes.

## Data Availability

The data used to support the findings of this study are included within the article.
